# Cost-effectiveness of newer technologies for the diagnosis of *Mycobacterium tuberculosis* infection in Brazilian people living with HIV

**DOI:** 10.1038/s41598-020-78737-w

**Published:** 2020-12-11

**Authors:** Ricardo E. Steffen, Marcia Pinto, Afranio Kritski, Anete Trajman

**Affiliations:** 1 Instituto de Medicina Social, Universidade do Estado do Rio de Janeiro, Rio de Janeiro, Brazil; 2 Departamento de Medicina, Pontifícia Universidade Católica do Rio de Janeiro, Rio de Janeiro, Brazil; 3 Instituto Nacional de Saúde da Mulher, da Criança e do Adolescente Fernandes Figueira, Fiocruz, Rio de Janeiro, Brazil; 4 Programa de Pós-Graduação em Clínica Médica, Universidade Federal do Rio de Janeiro, Rio de Janeiro, Brazil; 5 TB International Centre, McGill University, Montreal, Canada

**Keywords:** Health care economics, Infectious diseases

## Abstract

Tuberculosis is the leading cause of death among people living with HIV (PLH). Preventive tuberculosis therapy reduces mortality in PLH, especially in those with a positive tuberculin skin test (TST). New, more specific technologies for detecting latent tuberculosis infection (LTBI) are now commercially available. We sought to analyse the cost-effectiveness of four different strategies for the diagnosis of LTBI in PLH in Brazil, from the Brazilian public health care system perspective. We developed a Markov state-transition model comparing four strategies for the diagnosis of LTBI over 20 years. The strategies consisted of TST with the currently used protein purified derivative (PPD RT 23), two novel skin tests using recombinant allergens (Diaskintest [Generium Pharmaceutical, Moscow, Russia] and EC [Zhifei Longcom Biologic Pharmacy Co., Anhui, China]), and the QuantiFERON-TB-Gold-Plus (Qiagen, Hilden, Germany). The main outcome was cost (in 2020 US dollars) per quality-adjusted life years (QALY). For the base case scenario, the Diaskintest was dominant over all other examined strategies. The cost saving estimate per QALY was US $1375. In sensitivity analyses, the Diaskintest and other newer tests remained cost-saving compared to TST. For PLH, TST could be replaced by more specific tests in Brazil, considering the current national recommendations.

## Introduction

Tuberculosis is the leading cause of death from an infectious disease globally, including in people living with HIV^1^. The single most important strategy to control the epidemics is prevention of the disease through treatment of latent tuberculosis infection^2^. In people living with HIV, latent tuberculosis infection treatment reduces by 64% the risk of developing active disease for those with a positive TST^3^. The use of antiretroviral treatment has additive benefits to latent tuberculosis infection treatment in reducing both the incidence of active disease and overall mortality, with a lasting protective effect of 5 years^4,5^. The 2018 declaration of the United Nations high-level meeting^6^ on the fight against tuberculosis, the first dedicated to one disease, commits to providing treatment for latent tuberculosis infection to at least 6 million people living with HIV by 2022. While less than 18% of the overall population needing treatment for latent tuberculosis infection complete it—mainly through losses in several steps of the cascade-of-care of latent tuberculosis infection^7^—the number of people living with HIV that were provided with treatment for latent tuberculosis infection has increased recently, from 1.8 million in 2018 to 3.5 million in 2019^1^. Nevertheless, measures to minimize the impact of losses at each stage of care are important. Among the reasons identified across different countries and settings, pre-treatment losses, such as access to diagnostic tests for latent tuberculosis infection detection are an important bottleneck^7^.

Recognizing the high risk for tuberculosis among people living with HIV, the World Health Organization (WHO), since 2018^8^, recommends treatment of latent tuberculosis infection for people living with HIV in tuberculosis high-burden countries regardless of testing results. However, in Brazil, guidelines are different from WHO’s recommendations. In 2018, the Ministry of Health has adopted a distinctive policy regarding treatment of latent tuberculosis infection in people living with HIV: those with CD4+ cell counts 350 cells/μl or less should start treatment immediately and those with counts over 350 cells/μl should be tested for latent tuberculosis infection^9^. The current standard for latent tuberculosis infection diagnosis in the public health system is TST with PPD RT 23. This policy is based on systematic reviews and large studies^3,5,10,11^ that have repeatedly shown that benefit from treatment is higher in those with a positive tuberculin skin test (TST) compared with those with a negative TST result. Although more recent isolated studies in people living with HIV suggest that those with negative test results might be at greater risk of developing tuberculosis, and might also benefit from tuberculosis preventive therapy^4,12,13^, testing is still recommended for this high risk population in Brazil and other countries.

Currently, the TST with purified protein derivative (PPD RT 23) is the routine diagnostic test in most tuberculosis high burden countries, where 80% of cases are concentrated. Intermittent shortage of PPD^14^, the low specificity of the test in Bacille Calmette-Guérin (BCG) vaccinated populations^15^ and the cumbersome training necessary for TST has highlighted the need for adoption of newer, more specific tests. However, for the incorporation of new technologies to the public health system, local assessments of feasibility, acceptability, and cost-effectiveness are necessary^16^. We aimed to analyze the cost-effectiveness of newer latent tuberculosis infection diagnostic tests with the standard of care for diagnosing latent tuberculosis infection in people living with HIV in Brazil.

## Methods

### Setting and current policies

Brazil, a high-medium income country with the largest universal public health service, has one of the 30 highest tuberculosis and tuberculosis-HIV burden^1^. The tuberculosis and HIV programs provide diagnosis and treatment at no cost to patients. In 2018, data from the HIV care cascade in Brazil showed an estimate of 900,000 PLH, 66% of these on ART. There are no official or published data on the percentage of those who have completed one course of LTBI treatment but data from 2005 to 2009, from the THRio study, show that 48% have a CD4 cell count greater or equal to 350 cells/μl^17^. LTBI regimens currently recommended is 9 months of isoniazid or, for children under 15 years of age, adults over 50 years of age and those with liver disease, 4 months of rifampin. Three months of weekly doses of rifapentine and isoniazid has recently been approved but is not yet available in the public health system.

### Strategies

Four strategies were considered in this study. Three were based on skin tests, two using the recombinant ESAT-6 and CFP10 immunogens (Diaskintest [Generium Pharmaceutical, Moscow, Russia] and EC [Zhifei Longcom Biologic Pharmacy Co., Anhui, China]), and one using the tuberculin PPD RT 23 (Statens Serum Institut, Copenhagen, Denmark). The Diaskintest is currently used in Russia and commercially available in Russia, Kazakhstan and Ukraine^18^ and the EC immunogen is produced in China^19^. By substituting PPD with *Mycobacterium tuberculosis* specific antigens, they combine the operational advantages of the TST with the specificity of interferon-gamma release assays (IGRAs)^20^. The fourth strategy consists of an IGRA, the QuantiFERON-TB Gold Plus (QFT-Plus) test (Qiagen, Hilden, Germany)^21^. All new strategies were compared with TST, the standard care.

### Model structure

A state-transition Markov model was created, simulating a cohort of people living with HIV with a CD4^+^ cell count of 350 cells/μl or greater, for a time horizon of 20 years (20 annual cycles). The health states considered were (1) no latent tuberculosis infection, untested; (2) latent tuberculosis infection, untested; (3) active tuberculosis; (4) active tuberculosis, treated; (5) no latent tuberculosis infection, treated; (6) no latent tuberculosis infection, untreated; (7) latent tuberculosis infection, treated; (8) latent tuberculosis infection, untreated and (9) dead. The model was constructed using the software TreeAge Pro 2020 (Williamstown, MA).

The Markov model simulates long-term outcomes related to the natural history of latent tuberculosis infection. Progression to active tuberculosis, reinfection, treatment of latent tuberculosis infection and mortality from tuberculosis or other causes were considered. A proportion of people living with HIV with active tuberculosis are hospitalized and at increased risk of death. Screening for latent tuberculosis infection is offered to people living with HIV with a CD4^+^ cell count of 350 cells/μl or greater. Those with a positive test result are invited for a chest radiograph. Treatment is then recommended for those with latent tuberculosis infection. Latent tuberculosis infection is defined as contacts with a normal chest radiograph and a positive test result in the absence of signs or symptoms of active tuberculosis infection, following medical evaluation. When screening with a skin test, an individual may or may not return to have the test results interpreted (skin test not read). When screening with QFT-Plus, the result might be indeterminate. Treatment for latent tuberculosis infection was modeled as 9 months of isoniazid. During treatment, drug-induced liver injury may develop. Utility scores during tuberculosis treatment, development of drug-induced liver injury and post-tuberculosis treatment are decreased.

### Outcome

We calculated the incremental cost-effectiveness ratio (ICER) as incremental costs per quality-adjusted life year (QALY) gained for the three strategies compared to TST, discounting future costs and QALYs at 5% per year^22^. Costs and QALYs are accrued by individuals according to the length of time that they spend in each state of the model.

### Testing procedures and interpretation

The skin test procedure consists of using two tuberculin units of PPD RT 23 (TST) or recombinant tuberculin (Diaskintest or EC) on the volar aspect of the forearm, according to the Mantoux method^23^. The result is considered positive if there is an induration ≥ 5 mm 48–72 h after inoculation. The QFT-Plus procedure is based on the manufacturer's instructions using 1 ml aliquots of whole blood, incubated overnight with an antigen-free negative control, a phytohaemagglutinin-positive control and antigens to ESAT-6 and CFP-10^24^. When the result is indeterminate, the test is repeated once.

### Model parameters

#### Effectiveness data

The accuracy of QFT-Plus and PPD-based TST was estimated from meta-analyses^15,25,26^. The sensitivity and specificity of the two recombinant tuberculin proteins were considered to be similar to QFT-Plus, as the same antigens are used and head-to-head studies show similar test-positivity rates^19,27,28^. Although no studies have been performed comparing the tests on people living with HIV, this similarity is likely to be maintained.

Utility valuations were based on previously published studies from other countries^29–31^. Utilities were measured using SF-6D or a disease specific tool which may have been obtained using either standard gamble or time trade off techniques. Quality of life of people living with HIV and latent tuberculosis infection was 0.76, assuming no opportunistic infections. Quality of life with treatment-related adverse events was 0.25 during the period with drug-induced liver injury or severe allergic reactions and 0.62 with active tuberculosis^32–34^. Age-specific all-cause mortality rates in people living with HIV were taken from Brazilian specific data^35^, with increasing mortality rates as the cycle stages progressed.

#### Cost data

Costs were estimated from the perspective of the Brazilian Unified Health System (SUS) and from secondary sources^36^. The economic analysis included the following costs: diagnostic tests, outpatient visits, blood work, including liver function tests in case of drug-induced liver injury, chest radiograph, treatment of latent tuberculosis infection with 9 months of isoniazid, treatment of tuberculosis with isoniazid, rifampicin, pyrazinamide, and ethambutol, hospitalization due to tuberculosis and drug-induced liver injury leading to hospitalization. Costs were estimated from SUS National Procedures Table published elsewhere^36^. Hospitalization costs were based on the hospital reimbursement system used in Brazil, a process akin to diagnosis-related group reimbursement systems, for tuberculosis or drug-induced liver injury. Consumables costs for the diagnostic tests were based on the standard test procedures and from previous studies^37^. Costs of test kits were based on market value provided by the manufacturer, adjusted to 2020 Brazilian reais (R$) and then converted to 2020 US dollars using the annual average rate (US$ [rate US$1 = R$4.50]), according to the method described by Turner^38^. Societal costs (indirect costs, loss of productivity or cost of death) were not included.

#### Sensitivity analyses

One-way sensitivity analysis was carried out on all model inputs. Two-way sensitivity analysis was carried out on specific inputs with greatest impact on the one-way sensitivity analysis. To account for parameter uncertainty, we also performed a probabilistic sensitivity analysis in a Monte Carlo simulation with 10,000 iterations. The uncertainty in clinical probabilities and accuracies were assumed to have a beta distribution and a gamma distribution for costs. The generated distributions around costs, QALYs and the incremental cost-effectiveness ratio are shown using median and 95% uncertainty ranges (UR).

### Willingness to pay threshold

A cost-effectiveness acceptability curve was constructed, displaying the probability that the intervention is cost-effective from a range of willingness to pay threshold values. As Brazil does not have a willingness to pay threshold, a value of US $7544 per QALY, as proposed by Woods et al.^39^, was considered.

### Model assumptions

A number of assumptions were required to develop a feasible model. The cohort undergoes the routine procedures for people living with HIV according to national guidelines. All people being assessed for latent tuberculosis infection are people living with HIV with a CD4^+^ cell count of 350 cells/μl or greater. Those with a negative result will retest within 6–12 months. Those with a positive test result are then screened for active tuberculosis and undergo chest radiography and physical examination. The chest radiograph and physical examination are 100% accurate when ruling out the initial diagnosis of active tuberculosis. Individuals with a second indeterminate result on the QFT-Plus test are at the same risk of developing active tuberculosis as those with a negative result. All people with initial active tuberculosis accept treatment. Patients who complete tuberculosis treatment are considered cured. People who do not adhere to treatment for latent tuberculosis infection do not develop drug-induced liver injury. Treatment for latent tuberculosis infection without development of drug-induced liver injury did not decrease quality of life. Nonadherence to treatment due to adverse reactions occurred in the first month and conferred no partial protection. No health-related quality of life loss is experienced by people with latent tuberculosis infection. Progression from latent tuberculosis infection to active tuberculosis was simulated assuming a constant activation rate over time. Individuals with resolved active tuberculosis have an annual probability of relapse, with subsequent activation having the same probability as the initial episode. To account for waste in cost estimates, we considered the stability of newer recombinant allergens to be equal to that of PPD RT 23.

## Results

**Figure 1:**
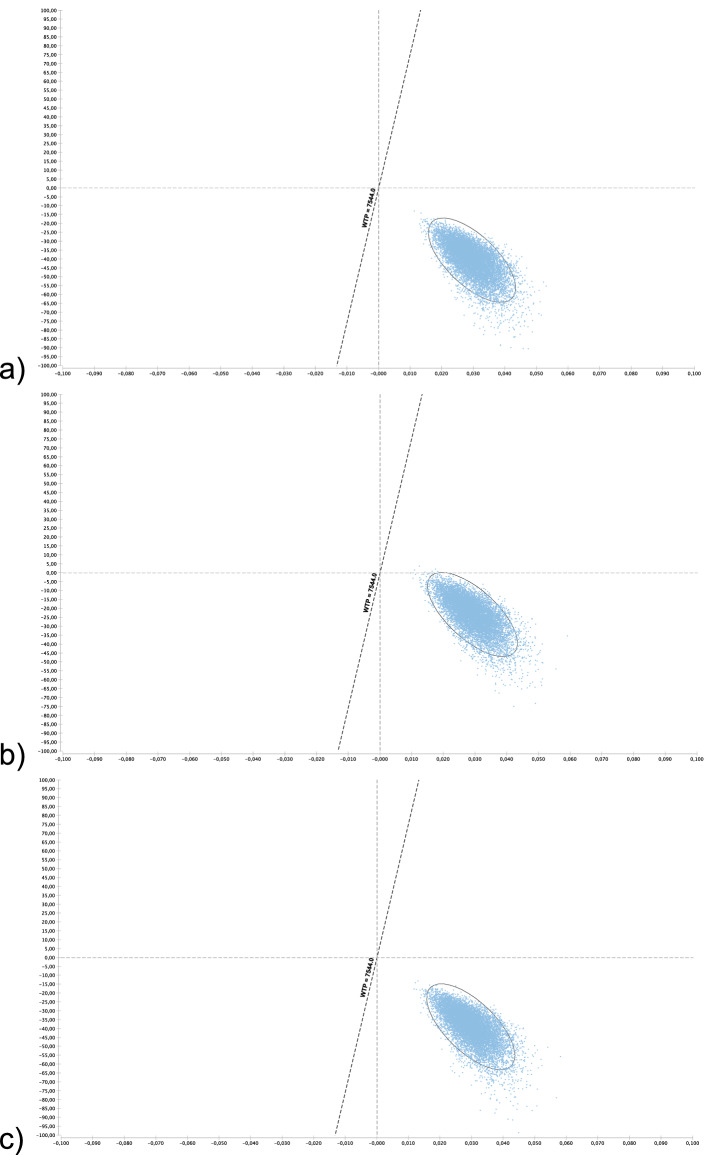
(**a**–**c**) Incremental cost effectiveness scatter plot of (**a**) Diaskintest vs. TST; (**b**) QFT-Plus vs. TST; (**c**) EC skin test vs. TST. Costs in 2020 US$ and effectiveness in QALYs. The graphs show the scatter plot of the resulting incremental cost-effectiveness ratio of 10,000 Monte Carlo simulations for the different strategies compared with tubeculin skin test. The diagonal line represents the willingness to pay threshold of US $7544. Values on the right lower quadrant are cost saving (less costly and more effective). The X-axis is scaled from − 0.1 to 0.1 QALY and Y-axis from − 100 to 100 US$. *TST* tuberculin skin test, *QFT-Plus* QuantiFERON-TB Gold Plus.

**Figure 2:**
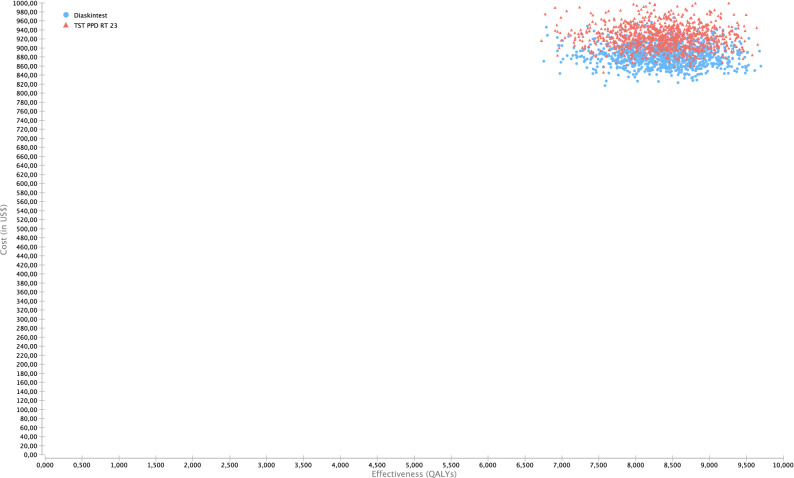
Cost-effectiveness scatter plot of Diaskintest versus TST. This figure shows a scatter plot of 10,000 Monte Carlo simulations for the total costs (in 2020 US$) and total effectiveness in quality-adjusted life years of the TST and Diaskintest strategies. *TST* tuberculin skin test.

**Figure 3:**
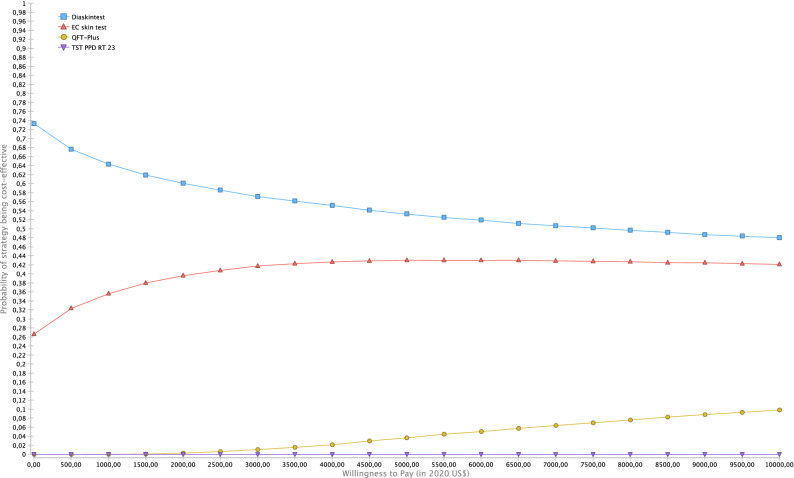
Cost-effectiveness acceptability curves (CEACs) of the different diagnostic strategies for latent tuberculosis infection. Cost effectiveness acceptability curves using the net-monetary benefit approach (10,000 Monte Carlo iterations) represent the probability (y-axis) that each strategy is more cost effective compared at the range of willingness to pay thresholds (US$ per quality-adjusted life-year [QALY]) on the x-axis. The curve is generated by repeating the procedure for various thresholds, with the threshold on x-axis and the probability to be cost effective on y-axis. Acceptability curves are presented here taking into account direct costs only. *CEAC* cost-effectiveness acceptability curve, *QALY* quality-adjusted life year, *QFT-Plus* QuantiFERON TB Gold Plus, *TST* tuberculin skin test, *US$* US dollars.

**Figure 4:**
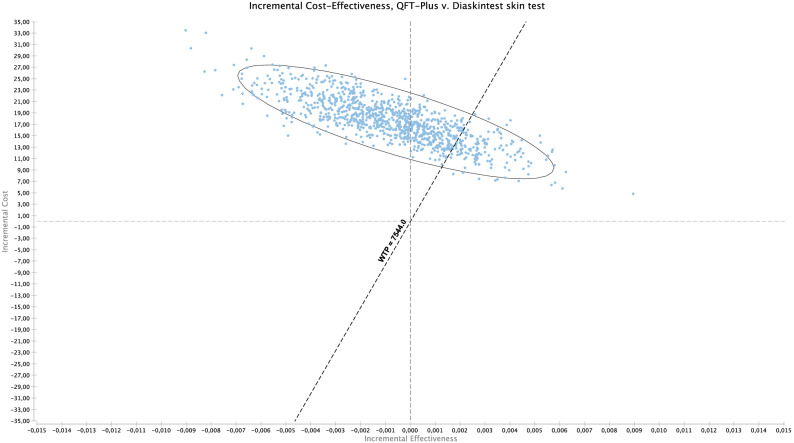
Scatter plot of QFT-Plus versus Diaskintest incremental cost-effectiveness ratio (ICER) in costs (in 2020 US dollars [US$]) per quality-adjusted life years (QALYs). This scatter plot shows 10,000 Monte Carlo iterations results of ICER of QFT-Plus versus Diaskintest. The diagonal line represents the willingness to pay threshold of US $7544. The elliptical line represents the 95% confidence interval. Values on the left upper quadrant are dominated. Values on the right upper quadrant are most costly and more effective. Values to the left of the diagonal line are above the willingness to pay threshold value. *QFT-Plus* QuantiFERON TB Gold Plus.

Model parameters for the base-case scenario are shown in Table 1. The base-case results show that (Table 2), compared with TST, all tests were cost-saving (Fig. 1a–c). The Diaskintest was cost saving at US $41 with an incremental gain of 0.03 QALYs, or US $1360 per QALY (95% UC $978–1948). The EC and QFT-Plus strategies were also cost saving at US $1283 (95% UC 904–2746) and US$771 (95% UC US $339–1336) per QALY, respectively. Overall, the TST strategy was dominated by all other strategies, since it had the highest cost, US $925, and a gain of 8.35 QALYs (Table 2). While Diaskintest strategy was the most cost saving, the result was sensitive to variations in the probabilistic sensitivity analysis (Fig. 2). Considering all strategies simultaneously, the Monte Carlo simulations show that, at a willingness to pay threshold of US $7544 per QALY, the Diaskintest had the highest net benefit in 50.4% of simulations, followed by the EC skin test (42.7%) and QFT-Plus (6.7%) (Fig. 3). Cost-effectiveness acceptability curves for each of the treatment strategies, showing the proportion of simulations where each strategy has the highest net benefit at different willingness to pay thresholds are also shown in Fig. 3. The results of the univariate sensitivity analyses are summarized in Table 3 and shown in the tornado diagram (see Supplementary Fig. S1). The impact of drug-induced liver injury risk on the final outcomes was negligible, based on the results of the univariate sensitivity analysis. When compared only with the Diaskintest strategy, QFT-Plus was cost-effective 17.5% of the time, at a willingness to pay of US $7544 (Fig. 4).

**Table 1:** Model parameters.

	Base-case	Range		PSA probability distribution	Source
		Low	High		
**Clinical and epidemiological parameters**					
Prevalence of LTBI	0.27	0.11	0.34	Beta	^17,48^
Probability of return to TST reading	0.88	0.65	0.97	Beta	^51^
Probability of starting LTBI treatment	0.82	0.74	0.97	Beta	^52^
Adherence to LTBI treatment (9 months)	0.63	0.38	0.86	Beta	^53–55^
Efficacy of LTBI treatment (9 months)	0.9	0.63	0.93	Beta	^56,57^
Probability of DILI related to LTBI treatment	0.002	0.001	0.005	Beta	^55^
Probability of hospitalization due to DILI	0.00015	0.00010	0.00020	Beta	^58^
Probability of death by DILI	0.00001	0.000001	0.0003	Beta	^58^
Progression from LTBI to TB, no treatment	0.08	0.04	0.11	Beta	^59^
**Test parameters**					
TST (PPD-RT23) sensitivity in PLH	0.61	0.5	0.86	Beta	^25^
TST (PPD-RT23) specificity in PLH	0.59	0.46	0.82	Beta	^15^
Diaskintest sensitivity in PLH	0.61	0.5	0.86	Beta	^19^
Diaskintest specificity in PLH	0.93	0.86	0.98	Beta	^16^
EC skin test sensitivity in PLH	0.61	0.5	0.86	Beta	^27^
EC skin test specificity in PLH	0.93	0.86	0.98	Beta	^27^
QFT-Plus sensitivity in PLH	0.61	0.5	0.86	Beta	^25,26^
QFT-Plus specificity in PLH	0.93	0.86	0.98	Beta	^26^
Probability of indeterminate QFT-Plus	0.04	0.01	0.07	Triangular	^25^
**Cost estimates (in 2020 US$)**					
LTBI diagnosis					
Initial medical visit	6.22	3.11	9.33	Gamma	MOH/SIGTAP
Chest radiograph	4.04	2.02	6.06	Gamma	MOH/SIGTAP
Total	**10.26**	**5.13**	**15.39**		
Active TB diagnosis					
Initial medical visit	6.22	3.11	9.33	Gamma	MOH/SIGTAP
Chest radiograph	4.04	2.02	6.06	Gamma	MOH/SIGTAP
Sputum smear	3.14	1.57	4.71	Gamma	MOH/SIGTAP
Total	**13.40**	**6.70**	**20.1**		
Active TB Treatment with DOT^a^	853.63	426.82	1280.45	Gamma	^60^
LTBI Treatment (9 months)	107	53.5	160.5	Gamma	^52^
Drug-induced liver injury					
Costs of hospitalization	432.88	216.44	649.32	Gamma	MOH/SIGTAP
Medical visit	6.22	3.11	9.33	Gamma	MOH/SIGTAP
Complete blood count	2.56	1.28	3.84	Gamma	MOH/SIGTAP
Liver enzymes	1.71	0.85	5.13	Gamma	MOH/SIGTAP
Total	**443.37**	**221.68**	**667.62**		
Diagnostic tests					
QFT-plus					
Human resources^b^	2.24	1.48	2.54	Gamma	^37^
QFT-Plus test kit	15.90	7.95	23.85	Gamma	Market value
Consumables^c^	1.81	1.31	1.97	Gamma	^34^
Equipment	1.07	1.07	1.60	Gamma	^34^
Skin tests					
Human resources^b^	2.12	1.48	2.54	Gamma	^34^
PPD RT23 2UT/1.5 ml	3.78	1.89	5.67	Gamma	Market value
Diaskintest	1.43	0.67	2.01	Gamma	Market value
EC	6.00	3.00	9.00	Gamma	Market value
Consumables	1.31	1.31	1.97	Gamma	^34^
Equipment	0.04				
Overall diagnostic test costs					
TST PPD RT23 2UT/1.5 ml	7.26	3.81	11.43	Gamma	Market value
Diaskintest	4.81	2.46	7.36	Gamma	Market value
EC skin test	9.47	4.74	14.22	Gamma	Market value
QFT-Plus	21.02	10.51	31.53	Gamma	Market value
Cost of death^d^	365.8	216.44	650.59	Gamma	MOH/SIGTAP
Discount rate	0.05	0	0.07	Triangular	^22^
Utility scores					
Latent TB infection, no opportunistic infection	0.76	0.57	0.91	Beta	^32–34^
HIV-infected, with active TB	0.62	0.45	0.75	Beta	^32–34^
Drug-induced liver injury	0.25	0.125	0.375	Beta	^32–34^
HIV-infected, recovered after TB treatment	0.76	0.38	1	Beta	^32–34^

*TB* tuberculosis; *PLH* people living with HIV; *TST* tuberculin skin test; *DILI* drug-induced liver injury; *QFT-Plus* QuantiFERON TB Gold Plus; *DOT* directly observed therapy; *MOH* Ministry of Health; *SIGTAP* table of procedures, medications, and orthotics, prosthetics, and special materials from the SUS; *PPD* purified protein derivative.

^a^DOT costs based on of 5 weekly visits during the intensive phase (first 2 months) and twice weekly during the continuation phase (remaining 4 months).

^b^Nursing staff time (for TST only), laboratory technician time (for QFT-Plus only).

^c^Gloves, cotton, alcohol, syringes with needles, box for syringes, thermic box and ice bag.

^d^Cost of death by based on hospitalization costs for pulmonary diseases.

**Table 2:** Strategy rankings—all referencing common baseline (in US$ 2020).

Strategy	Cost	Incremental cost	QALY	Incremental QALY	ICER	C/E
Diaskintest	884.70		8.386			105.50
EC skin test	886.60	1.90	8.386	0	(Undefined)	105.73
QFT-Plus	902.10	17.40	8.385	− 0.00055	− 31,415.80	107.59
TST PPD RT 23	925.50	40.80	8.356	− 0.02967	− 1,375.09	110.76

*QALY* quality-adjusted life year, *ICER* incremental cost-effectiveness ratio, *QFT-Plus* QuantiFERON TB Gold Plus, *TST* tuberculin skin test, *PPD RT 23* purified protein derivative, *C/E* cost effectiveness, *US$* US dollars.

**Table 3:** Summary of univariate sensitivity analyses (TST with PPD RT 23 versus Diaskintest).

Variable description	Variable low	Base	Variable high	Impact	Low	High
Prevalence LTBI	0.11	0.22	0.33	Increase	2575.60	0
Probability of returning to TST reading	0.71	0.88	0.97	Increase	− 2016.46	0
TST with PPD RT 23 specificity	0.72	0.59	0.91	Decrease	− 2256.90	− 1428.58
Cost per Diaskintest	9.48	4.82	15.56	Increase	− 1218.05	− 1013.16
Probability of starting LTBI treatment	0.55	0.82	0.95	Increase	− 1480.34	− 1345.55
Cost per TST with PPD RT 23	6.95	7.26	9.53	Decrease	− 1451.59	− 1364.64
Diaskintest specificity	0.86	0.93	0.98	Increase	− 1396.41	− 1364.55

*LTBI* latent tuberculosis infection, *TST* tuberculin skin test, *PPD RT 23* purified protein derivative.

## Discussion

This study compared the cost-effectiveness of newer, allegedly more specific skin tests (Diaskintest and EC) and an interferon-gamma release assay (QFT-Plus) against TST for the diagnosis of latent tuberculosis infection in people living with HIV. The main finding is that the Diaskintest is cost saving. QFT-Plus was more costly and equally effective. The TST was dominated by all the other strategies, since it identifies more subjects as eligible for preventive treatment (false positive test results), a result of the low specificity of TST^15^. The resulting loss effectiveness is driven by reduction in QALYs of more subjects developing drug-induced liver injury overall. Despite the greater number of people TST identifies as eligible for treatment, it predicts the same number of progressors (highest number needed to treat) as all the other strategies. A similar Markov simulation study^40^, performed in rheumatoid arthritis patients undergoing latent tuberculosis infection screening before starting tumor necrosis factor alpha antagonist therapy, found that in the base case analysis, QuantiFERON Gold In-Tube screening was less costly and more effective than the TST in populations regardless of BCG vaccination.

In the absence of a highly specific diagnostic test for latent tuberculosis infection in a population with a high risk of progression, safe and inexpensive treatment is necessary so that a larger proportion of tested people can accept and finish treatment. The choice of a low specificity for TST, however, might not be applicable to our setting, since it is based on a general misconception that BCG vaccination has a high impact on TST false positive results^41^. While this is true when subjects are vaccinated after 2 years of age, it is unlikely that a TST-positive test in a person living with HIV contact, BCG-vaccinated at birth, results from the vaccination rather than from latent tuberculosis infection. To account for this uncertainty in TST specificity, sensitivity analysis was performed with scenarios where TST had a higher specificity. The uncertainty surrounding other parameters, particularly modest differences in test costs and accuracy, coupled with the small incremental gains in QALYs, however, rendered the Diaskintest and EC skin tests as the favorable option. Additionally, when compared only with the Diaskintest, QFT-Plus was a cost-effective option 17.5% of the time in the Monte Carlo simulations (Fig. 3). Given the assumed similar sensitivity and specificity of the new tests, final effectiveness measures do not differ greatly, resulting in modest incremental QALYs. In the sensitivity analyses, the main drivers of differences in effectiveness between QFT-Plus and the skin tests were the probability of the patient returning for the skin test follow-up reading and the rate of indeterminate QFT-Plus results.

The modest incremental QALYs are common in cost-effectiveness analyses of strategies for the diagnosis of latent tuberculosis infection^42^. This is usually a result of the high number of persons needed to treat to avoid one case of tuberculosis, compounded by the small loss in QALY due to tuberculosis treatment and the risk of drug-induced liver injury. Other cost-effectiveness studies of testing and treatment for latent tuberculosis infection using simulation models have estimated an incremental QALYs of 0.0010 for TST and 0.0017 for IGRA, when compared to no testing^42^.

A recent meta-analysis^43^ has found that the incidence rate ratio of tuberculosis for people with a positive result versus negative result was 11-fold among people living with HIV, both for IGRA and TST. Given their similar predictive abilities, the adoption of latent tuberculosis infection diagnostic tests should consider other aspects, such as feasibility of adoption, costs with training, laboratory and clinical infrastructure needs, dependency on foreign suppliers for reagents and test kits, among others. Although the WHO recommends the treatment of latent tuberculosis infection in people living with HIV regardless of testing in resource-limited settings (WHO), the cost-effectiveness of this strategy was not included, since in Brazil testing is still recommended for those with a CD4^+^ cell count of 350 cells/μl or greater^44^. Additionally, the main focus of this study was to compare different, novel diagnostic strategies to the standard diagnostic procedure. Therefore, the evaluation of inexpensive, more specific alternatives to TST, are welcome. Access to a reliable supply of newer, more specific recombinant skin tests requiring little infrastructure to implement could provide important alternatives to testing this high risk population. Given the limited number of studies on the accuracy and predictive value of the newer skin tests, it is reasonable to infer that their predictive value will be similar to IGRAs, based on what we know about tests that use ESAT-6 and CFP-10 antigens^28,45^. Still, more validation studies and studies on their predictive ability are necessary, particularly in high risk populations. Also, at a national level, budget impact analysis and implementation studies are critical if scale-up and impact are to be considered^22^.

Although the Diaskintest was cost-saving over all other interventions, its net savings relies heavily on their final costs, which in turn is largely susceptible to fluctuations in exchange rates and optimization of supply chains for reagents. Aspects such as storage requirements, number of doses per vial and stability of reagents after opening the vials can have great impact on final test costs and, consequently, its cost-effectiveness. We considered the stability of newer recombinant reagents to be equal to that of PPD RT 23. Additionally, the incorporation of these tests into latent tuberculosis infection treatment programs could provide a stimulus for the local development of novel tuberculin skin tests and foster competition to supply regional demands. Another advantage over IGRAs is the lack of implementation costs, since it has been the standard practice in Brazil for years. The results of this study contrast with the results of previous studies in the Brazilian scenario^36,37^, where TST was found to have lower costs when compared with QFT-GIT. The outcome considered, however, was the number of tuberculosis cases averted, and did not take into account the loss in QALYs due to additional patients undergoing latent tuberculosis infection treatment. Also, the time horizon considered was only 2 years, and the population had significantly lower risk of progressing to active disease, since they were not immunocompromised. Additionally, costs of IGRA test kits are significantly lower today than they were at the time of previous studies.

The use of QFT-Plus, however, provides some additional advantages. Given that it is also cost saving when compared with TST, if implemented along a structured HIV treatment program, the operational advantages could make it an attractive option. Among the well-established advantages of QFT-Plus are: (1) it does not require an additional visit for test reading; (2) it provides an objective result, eliminating the interrater variability of skin tests; and (3) it can be implemented along with other routine blood test collection (i.e. CD4^+^ cell count) among people living with HIV in antiretroviral treatment follow-up visits.

This study has a few limitations. First, it assumed that testing for latent tuberculosis infection in people living with HIV is already cost-effective when compared to no screening, so the cost-effectiveness of the alternate strategy of tuberculosis symptom screening with no latent tuberculosis infection testing—as recommended by WHO—was not included. Previous studies have already established that targeted testing in this population^36,46–48^ is highly cost-effective in different settings. In addition, secondary tuberculosis transmission was not considered, due to the significant uncertainty that this variable can bring to the model when a 20-year time horizon is considered. Drug resistant tuberculosis strains were also not considered due to its modest prevalence in Brazil, despite the high treatment default rates^49^. Utility values were derived from studies performed in different settings and sensitivity analyses were performed to account for this uncertainty. The generalization of the study to other countries is limited, since health system structure, financing and salaries as well as other important parameters such as latent tuberculosis prevalence and annual risk of infection are widely variable. Finally, not all spectrum of TB was considered (incipient and sub-clinical disease)^50^, as there are currently no testing or treatment implications for other TB status.

One of the key strengths of the study is that it clearly shows that all the other strategies are dominant over the TST PPD RT 23 strategy, already shown to be cost-effective in this population^37,47^. It is also the only study, as far as we know, that has considered the costs and effectiveness of newer recombinant skin test, along with QFT-Plus, compared to the traditional TST. Additionally, this study used QALYs as an outcome measure which can capture not only loss in quality of life due to adverse events during the treatment for latent tuberculosis infection, but also years of life gained. This study also considered parameters comparable to previous studies evaluating the cost utility of screening high-risk populations for latent tuberculosis infection^47^. Finally, it provides additional alternatives to the QFT-Plus, namely the recombinant skin tests, which could combine the advantages of the traditional TST and the accuracy of QFT-Plus.

Health decision makers do not base their decisions exclusively on economic analyses. Other aspects, such as ease of implementation, equity, easy access to supplies and possibility of scale-up, are equally important.

## Conclusion

While the Diaskintest was the dominant strategy, this conclusion is still based on several assumptions. In the Brazilian scenario, considering the current recommendations, using any of the newer skin tests (Diaskintest or EC) or QFT-Plus is cost saving when compared to TST. Having additional options for the diagnosis of latent tuberculosis infection should contribute to eliminating the PPD RT 23 shortages, increase the competition among skin test manufacturers, decrease prices and foster innovation in latent tuberculosis infection diagnostics. However, additional studies, including budget impact analysis and implementation studies in Brazil, would be needed to recommend the incorporation of these new technologies.

## Supplementary Information

Supplementary Figure S1.

## Data Availability

All data generated or analysed during this study are included in this published article (and its “Supplementary Information” files).
