# Supplementary material for: Cost-effectiveness of newer technologies for the diagnosis of Mycobacterium tuberculosis infection in Brazilian people living with HIV
Source: Sci Rep. 2020 Dec 11;10:21823. doi: 10.1038/s41598-020-78737-w (PMC7733491; doi:10.1038/s41598-020-78737-w)
Supplement: Supplementary file 1 — Supplementary Figure S1. [file 41598_2020_78737_MOESM1_ESM.docx]

**Title: Cost-effectiveness of newer technologies for the diagnosis of *Mycobacterium tuberculosis* infection in Brazilian people living with HIV**

**Authors: Ricardo E. Steffen**, **Marcia Pinto**, **Afranio Kritski**, **Anete Trajman**

Correspondence should be addressed to Ricardo E. Steffen (e-mail: ricardo.steffen@gmail.com)

**Figure S1**

**Figure S1 – Tornado diagram for one-way sensitivity analysis of incremental cost-effectiveness ratio using US$ per additional quality-adjusted life-year.** Legend: Horizontal bars show the variation in incremental cost-effectiveness ratio (ICER; in US$ per QALY gained) with variation in the value of the parameter. Values of ICER below 0 indicate that the treatment is cost saving. Abbreviations: TST=tuberculin skin test; QALY = quality-adjusted life-year; ICER= Incremental cost-effectiveness ratio; TB=tuberculosis; TST=tuberculin skin test; LTBI=latent tuberculosis infection; QFT Plus=QuantiFERON-TB Gold Plus; DOT=directly observed treatment
